# Duodenal perforations secondary to a migrated biliary plastic stent successfully treated by endoscope: case-report and review of the literature

**DOI:** 10.1186/s12876-020-01294-z

**Published:** 2020-05-12

**Authors:** Xiaopeng Wang, Junwen Qu, Kewei Li

**Affiliations:** Department of Biliary- Pancreatic Surgery, Renji Hospital, School of Medicine, Shanghai Jiao Tong University, No. 160, Pujian Road, Pudong New Area, Shanghai, 200127 China

**Keywords:** Biliary stent, Migration, Duodenal perforation, Endoscope, Case report

## Abstract

**Background:**

Endoscopic retrograde biliary drainage (ERBD) is the most frequently performed procedure for treating benign or malignant biliary obstruction. Although duodenal perforations secondary to the biliary plastic stent are quite rare, they can be life-threatening. The treatment strategies for such perforations are diverse and continue to be debated.

**Case presentation:**

We report three cases of duodenal perforation due to the migration of biliary plastic stents that were successfully managed using an endoscope. The three patients were admitted on complaints of abdominal pain after they underwent ERBD. Abdominal computerized tomography (CT) revealed migration of the biliary plastic stents and perforation of the duodenum. Endoscopy was immediately performed, and perforation was confirmed. All migrated stents were successfully extracted endoscopically by using snares. In two of the three cases, the duodenal defects were successfully closed with haemostatic clips after stent retrieval, and subsequently, endoscopic nasobiliary drainage tubes were inserted. After the endoscopy and medical treatment, all three patients recovered completely.

**Conclusions:**

Duodenal perforations due to the migration of biliary stents are rare, and the treatment strategies remain controversial. Our cases and cases in the literature demonstrate that abdominal CT is the preferred method of examination for such perforations, and endoscopic management is appropriate as a first-line treatment approach.

## Background

Endoscopic retrograde cholangiopancreatography (ERCP) plays an important role in the diagnosis and treatment of a broad range of biliary and pancreatic diseases. However, this procedure is considered the most difficult and invasive endoscopic procedure with an associated complication rate of approximately 15% and mortality rate of 1% [1]. Endoscopic retrograde biliary drainage (ERBD) was first performed in 1979 and has since become a well-established palliative treatment for either benign or malignant biliary obstruction. The complications associated with endoscopic biliary stenting include stent migration, cholangitis, stent blockage, haemorrhage, perforation, and pancreatitis [2, 3]. Among these, stent migration is the most common complication. Distal or proximal displacement of a biliary stent occurs in 5–10% of patients with biliary stents [4]. However, gastrointestinal penetration or transmural perforation due to stent migration is rare, with an incidence of less than 1% [5]. Perforation due to biliary stent displacement can occur in the duodenum, jejunum, ileum, cecum, colon, and sigmoid colon [6–10].

Herein, we report three cases of duodenal perforation due to the migration of a plastic biliary stent encountered at Department of Biliary-Pancreatic Surgery, Shanghai Renji Hospital, since 2014 (Table 1.). Moreover, we review the current literature pertaining to the diagnosis, therapeutic options, and prognosis of stent-related duodenal perforation to provide a brief, updated overview of this devastating complication.

**Table 1:** Summary of the three cases in our hospital

Case, No.	Age/Sex	Diagnosis	No. of stents Diameter/length (manufacturer)	stent type (straight or pig-tailed)	Intervals from stent insertion to perforation (days)	Devices used for retrieving PS, closing perforation and biliary drainage	Perforation diameter (mm)	Postoperative hospitalization (days)	Outcome
1	72/M	CBDS and acute cholangitis	1 8.5 Fr/9 cm PS (Flexima™; Boston Scientific)	straight	33	Snare	3	19	recovery
2	84/M	AOSC and COPD	1 7 Fr/12 cm PS (Flexima™; Boston Scientific)	straight	3	Snare; Hemoclips; Nasobiliary drainage.	3	24	recovery
3	52/M	CBDS and acute cholangitis	1 8.5 Fr/9 cm PS (Flexima™; Boston Scientific)	straight	75	Snare; Hemoclips; Nasobiliary drainage.	3	12	recovery

abbreviations: *CBDS* Common bile duct stone, *AOSC* Acute obstructive suppurative cholangitis, *COPD* Chronic obstructive pulmonary disease, *PS* Plastic stent, *Fr* French

## Case presentation

### Case 1

A 72-year-old male suffering from right upper abdominal pain and fever for 1 day was admitted to the hospital. The patient was diagnosed with acute cholangitis and choledocholithiasis. An ERCP was performed on the patient by inserting an 8.5 Fr × 9 cm straight plastic biliary stent (FleximaTM, Boston Scientific). The patient was discharged after the symptoms were alleviated. Thirty-three days later, the patient complained of upper abdominal pain accompanied by fever. Abdominal computed tomography (CT) revealed that the biliary stent had migrated, with the distal end of the stent eroding the inferior wall of the third portion of the duodenum and protruding into the peritoneal cavity. A physical examination revealed mild tenderness in the right upper abdominal quadrant. Laboratory tests revealed that the patient’s white blood cell (WBC) count was 27.54 × 10^9^/L, percentage of neutrophils (N%) was 85%, haemoglobin level was 102 g/L, alanine aminotransferase (ALT) was 121 U/L, aspartate aminotransferase (AST) was 222 U/L, total bilirubin (TB) was 39.7 μmol/L, and direct bilirubin was 28.9 μmol/L; all other laboratory parameters were normal. A CT scan revealed no obvious signs of perforation.

ERCP was repeated. The endoscopic views revealed that the proximal tip of the previously placed plastic stent was in the bile duct, and the distal tip had penetrated the duodenal wall opposite the papilla. The plastic stent was successfully retracted from the duodenal wall and removed using rat-tooth forceps. The patient was managed with gastrointestinal decompression, antibiotics, and somatostatin and was discharged on day 19 post-procedure.

### Case 2

An 84-year-old man suffering from abdominal pain for 1 day was hospitalized. The patient underwent ERCP and endoscopic nasobiliary drainage (ENBD), in which a 7 Fr × 12 cm straight plastic biliary stent (FleximaTM, Boston Scientific) was inserted under a diagnosis of acute obstructive suppurative cholangitis and infectious shock. Three days later, the patient complained of aggravated abdominal pain. Abdominal CT revealed that the distal end of the biliary stent was protruding into the posterior peritoneum (anterior to the inferior vena cava).

ERCP confirmed perforation of the duodenum owing to the migrated biliary stent. The transmural defect was closed with three haemostatic clips after the migrated stent was extracted with a pair of foreign body forceps. Then, an ENBD tube was inserted. Postoperative fasting, fluid supplementation, anti-infection, and other symptomatic and supportive treatments were administered subsequently. The patient recovered completely and was discharged from the hospital 24 days after endoscopic management.

### Case 3

**Fig. 1:**
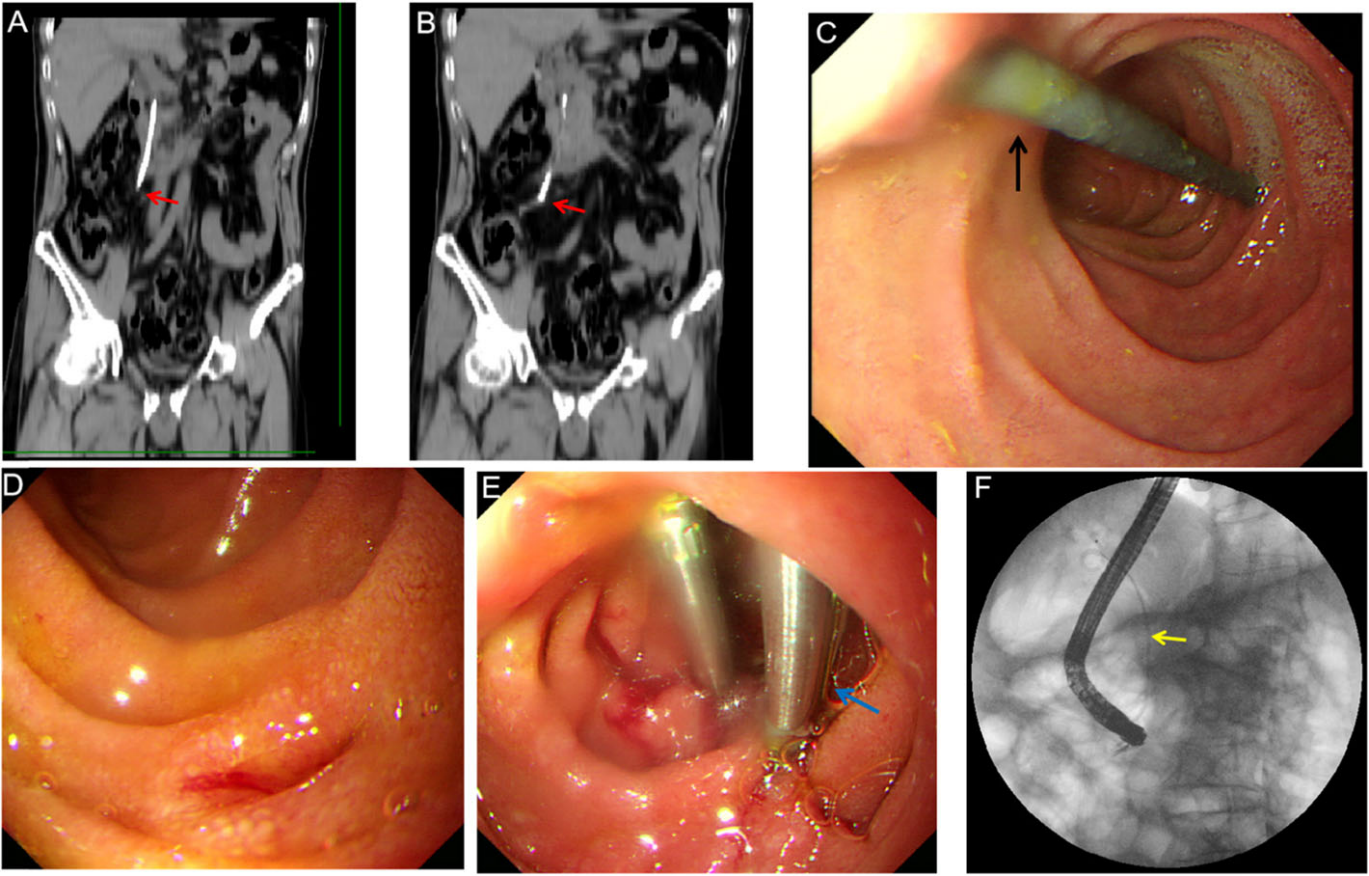
Duodenal perforation due to biliary plastic migration successfully managed by endoscope. **a** and **b** Coronal CT scan of the abdomen demonstrated that the biliary plastic stent (red arrow) had migrated and perforated through the duodenal wall. **c** Distal tip of biliary stent (black arrow) penetrated the duodenal wall which was confirmed during endoscope. **d** The migrated plastic stent was extracted by snare and perforation was detected during endoscopy. **e** The defect was successfully closed by hemostatic clips (blue arrow). **f** An endoscopic nasobiliary drainage (ENBD) tube (yellow arrow) was then inserted

A 52-year-old male suffering from upper abdominal pain and fever for 1 day was admitted to the hospital. He had a history of acute cholangitis and choledocholithiasis that had been managed with ERCP with the insertion of an 8.5 Fr × 9 cm straight plastic stent (FleximaTM, Boston Scientific) 75 days previously. A laboratory examination revealed an increase in the percentage of neutrophils (85.2%) and WBC (12.22 × 10^9^/L). An abdominal CT scan revealed that the distal end of biliary stent had penetrated the duodenal wall (Fig. 1a and b).

An ERCP was performed again. Initial endoscopic views revealed distal migration of the previously placed plastic biliary stent, with the distal tip of the stent penetrating the duodenal wall opposite the papilla (Fig. 1c). Stent retrieval was performed using a pair of foreign body forceps, and the perforation was closed using haemostatic clips (Fig. 1d and e). Additionally, an ENBD tube was placed in the common bile duct (Fig. 1f). The patient recovered completely and was dismissed from hospital 12 post-surgery.

## Discussion and conclusion

Currently, clinical classifications of ERCP-related perforations include the Stapfer classification (2000) [11], Howard classification (1999) [12], and Kim classification (2011) [13]. The Stapfer classification is the most widely used. However, none of the abovementioned classifications cover all types of ERCP-related perforations. For instance, intestinal wall perforations caused by bile duct stent migration, as mentioned in this paper, and perforation of the jejunum, ileum, cecum, colon, and sigmoid colon due to bile duct stent migration, as mentioned in other studies [6–10], cannot be included in the above classifications.

Endoscopically placed plastic biliary stents were first used for bile duct drainage in 1979. Subsequently, ERBD has become a widely accepted and well-established therapy for either benign or malignant biliary obstruction. The literature indicates that the complication rate for biliary stents can range from 8 to 10%, and the most common complication is proximal or distal stent migration [4]. Duodenal perforation secondary to distal stent migration has been well documented in the literature. However, owing to the low incidence of this serious and life-threatening complication (less than 1%) [5], preventive and therapeutic methods continue to be a matter of debate.

Stent-related duodenal perforations are mainly caused by distal biliary stent migration. Thus, it is very important to determine the risk factors of biliary stent migration. However, according to a few studies, thus far, the risk factors of stent migration have not been identified definitively. Kawaguchi et al. [14] demonstrated that the risk factors for biliary stent migration include straight-type stents, common bile duct diameter > 10 mm, stent duration > 1 month, and stent with large diameter (frequency of migration was significantly higher in cases with 10-Fr stents compared with that in cases with 7-Fr stents). Moreover, undergoing endoscopic sphincterotomy (EST) before stent placement and long biliary stent are considered risk factors for distal stent migration rather than proximal stent migration [4]. In addition to these risk factors, Arhan et al. [15] reported that biliary stent migration is more likely to occur in cases of benign biliary stenosis than in cases of malignant biliary stenosis. One reasonable explanation for this phenomenon is that local inflammation around the stent due to benign biliary strictures could be alleviated after biliary drainage. However, the growth of malignant tissues helps anchor the biliary stent tightly in cases of malignant strictures. In our cases, this type of perforation occurred between 3 and 75 days after ERBD, which demonstrated that stent-related duodenal perforation can occur regardless of stent duration. Among the three cases at our institution, two cases of distal stent migration were noted in patients with stent duration > 1 month. Moreover, according to our cases (Table 1) and the cases reported in the literature (Table 2), all migrated biliary stents are of the straight type, which are more prone to distal migration compared to pig-tailed biliary stents [14]. Double-pigtail biliary stents may exert an anti-migration effect by anchoring in the bile duct, and their pliable and soft plastic may prevent intestinal perforation due to stent migration. Especially, when treating patients with high risk factors for biliary stent migration, such as long stent duration, EST, common bile duct diameter > 10 mm, the use of pigtail stents is strongly recommended. Accordingly, periodic follow-ups and timely stent replacement are highly likely to prevent such perforations effectively. Moreover, the selection of suitable stents (pig-tailed, appropriate length, diameter etc.) and avoidance of unnecessary EST may have some preventive effect on such perforations.

**Table 2:** Summary of related cases found in the literature

Year	Sex/Age	Diagnosis	No. of stents Diameter/length (manufacturer) Stent type	Perforation-related symptoms and signs	Diagnostic method	Therapeutic method	Postoperative hospitalization (days)	Outcome
1993 [16]	F/55	Advanced GB cancer	1 10 Fr/15 cm biliary PS (Unmentioned) straight	Fever and chills	CT	Endoscopic removal	Death	Death from advanced GB cancer
2000 [17]	M/74	HCCA	1 10 Fr/15 cm biliary PS (Unmentioned) straight	Serve abdominal pain and vomiting	Endoscope	Endoscopic removal of PS and seal perforation by hemoclip	10	Improved
2006 [18]	M/55	Advanced HCCA with metastasis	1 Biliary PS (Unmentioned) straight	Abdominal distention and epigastric tenderness	ERCP	Endoscopic removal of PS	Death	Death from Intraoperative subcutaneous emphysema
2006 [19]	M/74	Periampullary carcinoma	1 7 Fr/10 cm biliary PS (Amsterdam) straight	Mild upper abdominal pain, vomiting and fever	Laparotomy	Laparotomy	Unmentioned	Recovery
2008 [20]	M/75	HCCA	2 Biliary PS (Unmentioned) straight	Unknown	Endoscope	Laparotomy	Unmentioned	Recovery
2008 [21]	F/52	HCCA	1 8.5 Fr/10 cm biliary PS (Unmentioned) straight	Fever and severe abdominal pain	Laparotomy	Laparotomy	Unmentioned	Recovery
2008 [22]	M/67	Periampullary carcinoma	1 10 Fr ST-2 biliary stent (Cook) straight	Rigid abdomen	CT	Laparotomy	Death	Death from postoperative complications
2012 [23]	M/55	Pancreatic ductal adenocarcinoma	2 Biliary PS and pancreatic stent (Unmentioned) straight	No symptoms	CT	Endoscopic removal	Unmentioned	Improved
2012 [24]	F/27	Benign Hilar biliary stricture	1 7 Fr/12 cm biliary PS (Unmentioned) straight	Upper abdominal pain	CT	Laparotomy	3	Recovery
2013 [25]	M/51	CBD stone	1 10 Fr/10 cm biliary PS (Unmentioned) straight	Deeply jaundiced and distended abdomen with diffuse tenderness and rigidity	CT	Laparotomy	12	Recovery
2014 [26]	M/67	Hilar biliary stricture after liver transplantation	1 8.5 Fr/10 cm biliary PS (Cotton-Leung; Wilson-COOK) straight	Fever	CT	Laparotomy	Unmentioned	Death from postoperative sepsis
2015 [27]	M/48	Hilar biliary stricture after liver transplantation	1 biliary PS (Unmentioned) straight	Abdominal pain and fever	CT	Endoscopic removal and seal perforation by over-the-scope clip	Unmentioned	Improved
2018 [28]	F/79	CBD stones and stricture	2 7 Fr/12 cm biliary PS; 10 Fr/15 cm biliary PS (Boston Scientific) straight	Nausea, vomiting, and abdominal pain	CT	Endoscopic removal by using Raptor™ grasping device and seal perforation by TTS endoclips	4	Recovery
2018 [29]	M/87	Acute cholangitis and CBD stones	1 10 Fr/15 cm biliary PS (Endo-FlexGmbH) straight	Diffuse abdominal pain of growing intensity	CT	Laparotomy	Unmentioned	Recovery
2019 [30]	M/71	Cholangiocarcinoma	1 8.5 Fr/12 cm biliary PS (Unmentioned) straight	Fever and abdominal pain	CT	Endoscopic removal and closure of perforation by over-the-scope clip. One 7 Fr/9 cm PS was positioned.	Unmentioned	Improved
2019 [31]	M/55	Post-cholecystec-tomy Biliary leak	1 10 Fr/10 cm biliary PS (CLSO, Cook) straight	Distended abdomen and diffuse abdominal tenderness	CT	Endoscopic removal of PS by using snare and closure of perforation by Hemoclips	Unmentioned	Unmentioned
2019 [31]	M/78	GB cancer	2 7 Fr/10 cm, 12 cm biliary PS (CLSO, Cook) straight	High fever and abdominal pain	CT	Endoscopic removal of stents by using rat-tooth forceps and closure of perforation by Hemoclips	Unmentioned	Improved
2019 [31]	M/72	Klaskin tumor, Bismuth type IIIa	1 10 Fr/12 cm biliary PS (Double Layer,Olympus) straight	Abdomen pain with tenderness	CT	Endoscopic removal of PS by using snare and closure of perforation by hemoclips and fibrin glue	Unmentioned	Improved
2019 [31]	F/84	Klaskin tumor, Bismuth type IIIa	1 10 Fr/12 cm biliary PS (CLSO, Cook) straight	Constant mild abdominal pain	CT	Endoscopic removal of PS by using snare and closure of perforation by hemoclips	Unmentioned	Improved
2019 [31]	F/73	GB cancer	1 10 Fr/15 cm biliary PS (CLSO, Cook) straight	Recurrent jaundice and abdominal pain	CT	Endoscopic removal of PS by using forceps forceps. Perforation was closed by Hemoclips. PTCD was performed	Unmentioned	Improved

abbreviations: *CBD* Common bile duct, *PS* Plastic stent, *Fr* French, *GB* Gallbladder, *HCCA* Hilar cholangiocarcinoma;

A distally migrated stent may cause intraperitoneal or retroperitoneal perforations. The symptoms are usually presented early and typically. In our cases, all patients presented with obvious abdominal pain in the early stages after perforation. Laboratory tests demonstrated a significant increase in WBC count and neutrophil percentage, accompanied by an increase in serum amylase to varying degrees. However, the diagnoses of both perforation and post-ERCP pancreatitis can be associated with abdominal pain accompanied by increased serum amylase. Thus, the diagnosis of perforation can be challenging and difficult, especially when perforation occurs early or is accompanied by pancreatitis post-ERCP. If a perforation is highly suspected, an abdominal CT should be the preferred imaging examination. The diagnosis of a stent-related duodenal perforation can be confirmed by CT findings of intraperitoneal or retroperitoneal free air, effusion, or duodenal wall penetration caused by the distal stent. Immediate recognition of perforations is very important for the selection of surgical intervention and achieving more favourable patient outcomes.

Presently, no relevant guidelines exist for preventing and managing duodenal perforations secondary to stent migration. Herein, we reviewed the extant literature and found that 20 cases involving stent-related duodenal perforation have been reported thus far (Table 2). Of these 20 cases, 8 cases were treated with surgery and 12 with endoscopic management. Different surgical methods, such as simple repair, duodenectomy, choledochojejunostomy, and gastric bypass, were selected depending on the specific conditions during the surgery. In the 12 cases of endoscopic management of duodenal perforation reported in the literature, all distally migrated stents were extracted directly by using foreign body forceps or snares. In 10 of these 12 cases, the perforated duodenal wall was successfully sealed with endoscopic clips or fibrin glue under observation with an endoscope. As shown in Table 2, surgical management was previously recognized as the main treatment modality for stent-related duodenal perforation. However, endoscopic stent removal and closure is currently justified for the management of such perforations, mainly because of the development of endoscopic techniques [31]. Undeniably, the treatment strategies for such perforations are increasingly tending toward minimally invasive and conservative procedures. Of the three cases in our hospital, in the first case, owing to our lack of experience, the only endoscopic treatment performed was removal of the displaced stent. In the other two cases, we inserted nasobiliary drainage after endoscopic removal of the displaced stents and closure of the duodenal perforations. In all the three cases, the patients recovered completely owing to effective endoscopic management. The length of hospitalization of the third patient was significantly shorter than those of the first two. Therefore, we believe that closure of the defect and bile drainage using ENBD tubes can reduce bile stimulation of the defects on the duodenal wall, which is conducive to create a good environment for the healing of perforated sites and hastening patient rehabilitation.

According to previous literature (Table 2), there were four deaths. Of these cases, two patients underwent surgical intervention and two received endoscopic management. The two patients who underwent surgical intervention died from postoperative infection. Of the two patients who underwent endoscopic management, one died from advanced gallbladder cancer 3 months after successful endoscopic management of the perforation, and the other patient died from intraoperative mediastinal emphysema, an extremely rare complication owing to air leakage from the defect in the intestinal wall. The use of minimal CO2 insufflation instead of air insufflation and reduction of procedure time could possibly have prevented this fatal complication. Moreover, unexplained dyspnoea, chest pain, and oxygen desaturation accompanied with abdominal distension during endoscopic repair of perforations must alert clinicians to this life-threatening complication. A diagnosis of pneumothorax can be further confirmed using fluoroscopy, chest CT scan, or X-ray combined with diminished breath sounds. According to a review of the reported literature [32], most patients with ERCP-related pneumothorax can be treated conservatively by means of nasogastric tube placement, chest drainage, and broad spectrum antibiotics. Early diagnosis and prompt management are important for optimising patient outcomes.

In our opinion, endoscopic management of duodenal perforations due to biliary stent migration has the following advantages: (i) The diameter of defect is small in most cases; thus, an endoscopic clip can effectively close the defect after removal of the stent under observation with an endoscope. (ii) Bile drainage plays an important role in the healing of duodenal defects secondary to biliary stent migration. ENBD is an effective technique for biliary drainage that can help prevent both bile-induced irritation of the defect and recurrence of biliary obstruction secondary to the primary disease after stent removal. (iii) Endoscopic treatment can reduce the degree of invasiveness and ensure faster recovery than laparotomy; however, it has higher requirements in terms of the endoscopist’s skill and experience. All three cases in our department were cured with endoscopic management, demonstrating that endoscopic treatment can often achieve ideal results for such perforations.

In conclusion, duodenal perforation caused by the migration of biliary stents is a rare complication. Abdominal CT scanning is the preferred imaging examination, and subsequent endoscopic treatment is feasible and effective.

## Data Availability

The datasets used and/or analysed during the current study are available from the corresponding author on reasonable request.

## Abbreviations

ERBD: Endoscopic retrograde biliary drainage
CT: Computerized tomography
ERCP: Endoscopic retrograde cholangiopancreatography
CBDS: Common bile duct stone
AOSC: Acute obstructive suppurative cholangitis
COPD: Chronic obstructive pulmonary disease
PS: Plastic stent
Fr: French
CBD: Common bile duct
GB: Gallbladder
HCCA: Hilar cholangiocarcinoma
SEMS: Self-expandable metal stents
WBC: White blood cell
N%: Percentage of neutrophils
ALT: Alanine aminotransferase
AST: Aspartate aminotransferase
